# Physical Performance Limitations in Adolescent and Adult Survivors of Childhood Cancer and Their Siblings

**DOI:** 10.1371/journal.pone.0047944

**Published:** 2012-10-17

**Authors:** Corina S. Rueegg, Gisela Michel, Laura Wengenroth, Nicolas X. von der Weid, Eva Bergstraesser, Claudia E. Kuehni, Swiss Paediatric Oncology Group (SPOG), R. Ammann, R. Angst, M. Beck Popovic, E. Bergstraesser, P. Brazzola, U. Caflisch, J. Greiner, M. Grotzer, H. Hengartner, T. Kühne, K. Leibundgut, F. Niggli, L. Nobile Buetti, H. Ozsahin, M. Paulussen, J. Rischewski, N. von der Weid

**Affiliations:** Bern; Aarau; Lausanne; Zurich; Bellinzona; Lucerne; St. Gallen; Zurich; St. Gallen; Basel; Bern; Zürich; Locarn; Geneva; Basel; Lucerne; Lausanne; 1 Swiss Childhood Cancer Registry, Institute of Social and Preventive Medicine, University of Bern, Bern, Switzerland; 2 Paediatric Hematology-Oncology Unit, Centre Hospitalier Universitaire Vaudois (CHUV), Lausanne, Switzerland; 3 Department of Oncology, University Children's Hospital Zurich, Zurich, Switzerland; Ospedale Pediatrico Bambino Gesù, Italy

## Abstract

**Purpose:**

This study investigates physical performance limitations for sports and daily activities in recently diagnosed childhood cancer survivors and siblings.

**Methods:**

The Swiss Childhood Cancer Survivor Study sent a questionnaire to all survivors (≥16 years) registered in the Swiss Childhood Cancer Registry, who survived >5 years and were diagnosed 1976–2003 aged <16 years. Siblings received similar questionnaires. We assessed two types of physical performance limitations: 1) limitations in sports; 2) limitations in daily activities (using SF-36 physical function score). We compared results between survivors diagnosed before and after 1990 and determined predictors for both types of limitations by multivariable logistic regression.

**Results:**

The sample included 1038 survivors and 534 siblings. Overall, 96 survivors (9.5%) and 7 siblings (1.1%) reported a limitation in sports (Odds ratio 5.5, 95%CI 2.9-10.4, p<0.001), mainly caused by musculoskeletal and neurological problems. Findings were even more pronounced for children diagnosed more recently (OR 4.8, CI 2.4–9.6 and 8.3, CI 3.7–18.8 for those diagnosed <1990 and ≥1990, respectively; p = 0.025). Mean physical function score for limitations in daily activities was 49.6 (CI 48.9–50.4) in survivors and 53.1 (CI 52.5–53.7) in siblings (p<0.001). Again, differences tended to be larger in children diagnosed more recently. Survivors of bone tumors, CNS tumors and retinoblastoma and children treated with radiotherapy were most strongly affected.

**Conclusion:**

Survivors of childhood cancer, even those diagnosed recently and treated with modern protocols, remain at high risk for physical performance limitations. Treatment and follow-up care should include tailored interventions to mitigate these late effects in high-risk patients.

## Introduction

Beyond survival, modern childhood cancer treatment strives to preserve long-term functionality and health-related quality of life [1]. While survival rates have reached 80% [2], [3], there is increasing evidence for short- and long-term adverse effects, including physical performance limitations [4], [5]. These can affect health-related quality of life, reduce educational and occupational achievements, impede healthy lifestyle and independent living, and interfere with social development, including interaction with peers, finding a partner and founding a family [6], [7].

There are two broad types of physical activity: a) participation in sports, and b) participation in activities of daily life [8]. The first type of activities (sports) is important for social integration with peers and for preventing or mitigating adverse late effects of childhood cancer, including cardiovascular disease, obesity, osteoporosis, or chronic fatigue [9], [10], [11]. The second type of activities (daily activities), is essential for being able to live independently and enjoy a good quality of life [6].

Only few studies from the US [6], [7], [12], [13], [14], [15] and one from the UK [16] investigated physical performance after childhood cancer. All focused on limitations of daily activities, using questions from the short form 36 (SF-36) or similar questions from the Behavioral Risk Factor Surveillance System Questionnaire (BRFSS) [7], [16]. They reported an increased likelihood of physical performance limitations in survivors compared to siblings or the general population with odds ratios ranging from 1.8–5.9 [12], [14], [15]. Survivors of bone tumors, brain tumors, and Hodgkin's lymphoma were most affected [12], [13], [14], [15]. Both the US and UK cohorts studied adult survivors diagnosed and treated decades ago (US 1970–1986 and UK 1940–1991) [17], [18].

We hypothesized that improvements in treatment over time have decreased the risk of physical performance limitations. Our study is the first to include recently diagnosed survivors (until 2003), and young survivors still in their adolescence. Moreover, this is the first study assessing also limitations in sports as well as the underlying causes. Together with daily activities this covers a broad spectrum of physical performance.

Using the population-based Swiss Childhood Cancer Survivor Study (SCCSS), our goal was to compare physical performance limitations in sports and daily activities of survivors and siblings, including recently diagnosed survivors. We tested whether these limitations varied by time period of diagnosis and assessed how they differed by type of cancer, treatments, and socio-demographic variables.

## Materials and Methods

### Ethics statement

Ethics approval was provided through the general cancer registry permission of the Swiss Childhood Cancer Registry (The Swiss Federal Commission of Experts for Professional Secrecy in Medical Research) and a non obstat statement was obtained from the ethics committee of the canton of Bern, stating that no additional ethics permission and no additional informed consent was necessary for the Swiss Childhood Cancer Survivor Study. All information regarding individuals from the Swiss Childhood Cancer Survivor Study was made anonymous to investigators prior to analysis.

### The Swiss Childhood Cancer Survivor Study (SCCSS)

The SCCSS is a population-based long-term follow-up study of all patients registered in the Swiss Childhood Cancer Registry (SCCR), diagnosed 1976–2003 at an age of 0–15 years, who survived ≥5 years [19]. The SCCR includes all children and adolescents in Switzerland diagnosed with leukemia, lymphoma, central nervous system (CNS) tumors, malignant solid tumors or Langerhans cell histiocytosis before age 21 years [20].

In 2007–2010 we traced all addresses of eligible survivors for the SCCSS and sent them an extensive questionnaire [19]. Non-responders received another questionnaire and were then contacted by phone. The questionnaires were similar to those of the US and UK childhood cancer survivor studies [17], [18], but we added questions on health behaviors and socio-demographic measures from the Swiss Health Survey 2007 [21] and the Swiss Census 2000 [22].

Siblings of survivors were recruited as a comparison group. In the questionnaire, survivors were asked to list their siblings. In 2010–2011 we asked survivors with siblings for consent to contact them and provide us with their address. Siblings received the same questionnaire as survivors, without questions relating to cancer history. Siblings, who did not respond, received the questionnaire again after 4–6 weeks but were not reminded by phone.

### Outcome measures: performance limitations

The questionnaire assessed two different types of performance limitations:

1) “Limitations in sports” were assessed by asking participants whether or not they had “any limitation in sporting activities”. If so, they were asked to describe the limitation in detail. Three pediatricians (CEK, EB, NXvdW) manually coded these answers into broad categories of medical conditions. When participants reported more than one problem, the most severe was used for analysis.
2) “Limitations in daily activities” were defined as low physical function in the SF-36 [23], [24]. The physical function score aggregates ten questions related to tasks of daily living, such as carrying groceries, climbing stairs, bending down, walking a certain distance, dressing or bathing. Raw scores were converted to T scores (mean = 50, SD = 10) according to age- and sex-stratified norm data from a public use-file from the German Federal Survey (N = 6964) [25]. For the logistic regression model we created a binary variable, using a natural cutoff value below the 5^th^ percentile of the distribution of the sibling population (score<45). Survivors below this cutoff were defined as “limited in daily activities”. We did sensitivity analyses with a cutoff at 10% below the siblings' distribution (score<50), which had been previously used in other studies [14], and at one standard deviation below the siblings' mean (score<47). Predictors from the regression model did not differ depending on the cutoff.

### Explanatory variables

Baseline demographic data and prospectively collected medical information on diagnosis and treatment of survivors was extracted from the Swiss Childhood Cancer Registry: gender, age at diagnosis, cancer diagnosis, cancer treatment, relapse, time since diagnosis, and age at survey. We used two explanatory variables assessed by questionnaire: migration background and parental education.

We used the International Classification of Childhood Cancer – 3^rd^ Edition [26] to classify diagnosis. For the descriptive analysis treatment modalities were assessed separately. For the regression models, treatment was hierarchically classified as surgery only, chemotherapy with or without surgery, radiotherapy with or without chemotherapy or surgery, and bone marrow transplantation (BMT). Participants were classified as having an immigrant background if they were not Swiss citizens since birth, not born in Switzerland, or had at least one parent who was not Swiss citizen. Parent's education was divided into three categories: primary (compulsory schooling only); secondary (including vocational training, teachers, technical and commercial schools etc.); and tertiary (including university and university of applied sciences) [22], [27].

### Statistical Analysis

Using Stata version 11.0, we analyzed data for all survivors and siblings aged ≥16 years at time of survey. Results from siblings were age and sex standardized for comparison.

First, we determined the proportion of survivors and siblings reporting a limitation for sports and the reasons for these limitations. Survivors and siblings were compared by logistic regression adjusted for age and sex. In a sensitivity analysis the regression model was also adjusted for family clustering [28].

Second, we described limitations in daily activities using the SF-36 physical function score and its single items. We compared mean scores of survivors and siblings using linear regression adjusted for age and sex. Again, we adjusted for family clustering in a sensitivity analysis.

Third, we tested whether results from step one and two differed between survivors diagnosed 1976–89 and 1990–2003, respectively. In a sensitivity analysis we did step one and two for survivors diagnosed in the last 5 years of our cohort only (1998–2003).

Finally, we used univariable and multivariable logistic regression models to identify predictors of both types of performance limitations in survivors, and likelihood ratio tests to calculate global p-values.

## Results

### Study population

We traced addresses of 1445 of 1552 eligible survivors (**Figure S1**). Of those, 1121 (78%) returned a questionnaire, 1038 (72%) the full-length questionnaire, and 83 (6%) an abbreviated version without questions on performance limitations. Participants (n = 1038), in comparison to non-participants (n = 514) (**Table** **1**), were more often female (48% vs. 37%; p = <0.001), aged 20–30 years (52% vs. 43%; p = 0.011), and treated with BMT (8% vs. 3%; p<0.001). They did not differ by type of cancer, age at diagnosis, time since diagnosis or treatment. Most survivors had suffered from leukemia (37%), lymphoma (19%) or a CNS tumor (13%), 67% had been treated with surgery, 84% with chemotherapy and 38% with radiotherapy of whom 167 (42.5%) received cranial irradiation. Of the 80 participants who had bone marrow transplantation (BMT), 48 (60%) were treated with autologous BMT and 30 (37.5%) with allogeneic BMT. Mean age at diagnosis was 7.7 years (SD 4.7) and mean time elapsed since diagnosis 18.2 years (SD 6.9). We received consent to contact 1293 siblings. Of those, 534 (41%) returned a questionnaire.

**Table 1:** Characteristics of the study population, comparing participants, non-participants and siblings of the current analysis.

	Survivor participants (n = 1038)		Sibling participants^a^ (n = 534)			Survivor non-participants^b^ (n = 514)		
	n	%^c^	n	%^c^		n	%^c^	p-value^d^
*Gender*								<0.001
Male	545	52.5	282	52.8		322	62.7	
Female	493	47.5	252	47.3		192	37.4	
*Age (years)*								0.011
<20	234	22.5	110	20.6		138	26.9	
–29.9	536	51.6	293	54.9		219	42.6	
30–39.9	228	22.0	111	20.8		133	25.9	
≥40	40	3.9	20	3.8		24	4.7	
*Migration background*								
None (Swiss)	795	76.6	454	85.1	n.a.^e^			
Germany, Austria, France	39	3.8	16	3.0				
Italy, Spain	87	8.4	33	6.1				
Other countries	117	11.3	31	5.9				
*Education of parents*								
Primary education	88	8.5	37	7.0	n.a.^e^			
Secondary education	726	69.9	342	64.1				
Tertiary education	160	15.4	85	15.9				
Unknown	64	6.2	70	13.0				
*Age at diagnosis (years)*								0.136
<5	372	35.8	n.a.^f^		202		39.3	
5–9.9	286	27.6			150		29.2	
≥10	380	36.6			162		31.5	
*Time since diagnosis (years)*								0.866
<10	134	12.9	n.a.^f^		63		12.3	
10–19.9	483	46.5			231		44.9	
20–29.9	351	33.8			184		35.8	
≥30	70	6.7			36		7.0	
*Diagnosis (ICCC-3)*								0.609
I Leukemia	383	36.9	n.a.^f^		169		32.9	
II Lymphoma	195	18.8			107		20.8	
III CNS tumor	132	12.7			67		13.0	
IV Neuroblastoma	45	4.3			20		3.9	
V Retinoblastoma	21	2.0			15		2.9	
VI Renal tumor	70	6.7			29		5.6	
VII Hepatic tumor	5	0.5			2		0.4	
VIII Bone tumor	42	4.1			16		3.1	
IX Soft tissue sarcoma	56	5.4			30		5.8	
X Germ cell tumor	30	2.9			18		3.5	
XI & XII Other tumor^g^	12	1.2			7		1.4	
Langerhans Cell Histiocytosis	47	4.5			34		6.6	
*Therapy* h								
Surgery	698	67.2	n.a.^f^		357		69.5	0.380
Chemotherapy	871	83.9			419		81.5	0.236
Radiotherapy	393	37.9			214		41.6	0.152
Bone Marrow Transplantation	80	7.7			14		2.7	<0.001
*Relapse*								0.041
yes	107	10.3	n.a.^f^		71		13.8	
No	931	89.7			443		86.2	

NOTE: Percentages are based upon available data for each variable.

Abbreviations: CNS, Central Nervous System; ICCC-3, International Classification of Childhood Cancer – Third Edition; n, number; n.a., not applicable.

a Age- and sex-standardized numbers and percentages are given for siblings.

b Non participants include: 107 without current address, 239 who did not respond, 85 who refused to participate, and 83 who answered an abridged questionnaire (**Supplementary Figure S1**).

c Column percentages are given.

d P-value calculated from chi-square statistics comparing survivor participants and survivor non-participants.

e Information derived from questionnaire survey is not available for non-responders.

f Information on former cancer disease is not applicable for siblings.

g Other malignant epithelial neoplasms, malignant melanomas and other or unspecified malignant neoplasms.

h Each person can have had several treatments.

### Limitations in sports

Overall, 96 (9.5%; 95% Confidence Interval (CI) 7.8–11.4) survivors reported a limitation in sports (**Table** **2**). Most limitations were caused by musculoskeletal problems (n = 43, 4.2%), followed by neurological problems (n = 27, 2.7%), and pain and fatigue syndromes (n = 7, 0.7%). Among those with limitations, 14 survivors (1.3% of 1038) reported a severe handicap such as dependence on a wheel chair. Twenty survivors (1.9%) reported more than one limitation. Type of limitation differed by type of diagnosis (**Table S1**). For example most survivors of CNS tumors suffered from neurological problems and survivors of bone tumors or lymphoma from musculoskeletal problems.

**Table 2:** Description of limitations in sporting activities and daily activities in survivors and siblings.

	Survivors			Siblings^a^					
Limitation in sporting activities									
*Medical conditions*	**N**	**%**	**95% CI**	**N**	**%**	**95% CI**	**OR** b	**95% CI**	**p-value** c
Musculoskeletal problems	43	4.2	3.2–5.7	3	0.5	0.2–1.2			
Neurological problems	27	2.7	1.8–3.9	2	0.3	0.1–1.1			
Pain and fatigue syndromes	7	0.7	0.3–1.4	0	0	-			
Weight and endurance problems	5	0.5	0.2–1.2	0	0	-			
Cardio-pulmonary problems	3	0.3	0.1–0.9	1	0.2	0.02–1.1			
Visual impairment	3	0.3	0.1–0.9	0	0	-			
Psychological problems	2	0.2	0.1–0.8	0	0	-			
Problem unknown	6	0.6	0.3–1.3	1	0.2	0.02–1.2			
**Total proportion**	**96**	**9.5**	**7.8–11.4**	**7**	**1.1**	**0.6–2.1**	**5.5** b	**2.9–10.4**	**<0.001**
Limitations in daily activities									
*Items of physical function score*	**N**	**%**	**95% CI**	**N**	**%**	**95% CI**	**Diff.** d	**95% CI**	**p-value** c
Vigorous activities	336	32.6^e^	29.8–35.6	91	17.0^e^	14.0–20.6			
Moderate activities	91	8.9^e^	7.3–10.8	14	2.7^e^	1.6–4.6			
Carrying groceries	82	8.0^e^	6.5–9.8	19	3.6^e^	2.3–5.7			
Climbing several flights of stairs	108	10.5^e^	8.8–12.6	21	3.9^e^	2.5-6.0			
Climbing one flight of stairs	34	3.3^e^	2.4–4.6	4	0.7^e^	0.3–1.9			
Bending down	124	12.1^e^	10.2–14.2	29	5.5^e^	3.7–8.2			
Walking more than 1 kilometer	98	9.5^e^	7.9–11.5	12	2.3^e^	1.2–4.3			
Walking several 100 meters	57	5.5^e^	4.3–7.1	5	0.9^e^	0.4–2.1			
Walking 100 meters	38	3.7^e^	2.7–5.0	4	0.8^e^	0.4–1.9			
Bathing or dressing	29	2.8^e^	2.0–4.0	4	0.7^e^	0.3–1.9			
**Physical function score (mean)**		**49.6** f	**48.9–50.4**		**53.1** f	**52.5–53.7**	**−3.3** d	**−4.5–2.1**	**<0.001**

Abbreviations: CI, Confidence Interval; Diff., Difference; N, Number; SF-36, Short Form 36; OR, Odds Ratio.

a Age- and sex-standardized numbers and percentages are given for siblings based on the marginal distribution in survivors.

b OR comparing survivors and siblings in a logistic model adjusting for age and sex.

c P-values calculated from regression models adjusting for age and sex.

d Coefficient comparing mean score in survivors and siblings from linear regression adjusting for age and sex.

e Proportion who indicated to be limited either a lot or a little in single items if the SF-36 physical function score.

f Mean of T-standardized physical function score of the SF-36 (23).

Among siblings, only 7 (1.1%; CI 0.6–2.1) reported a limitation in sports (**Table 2**). None was severely handicapped or reported more than one limitation. The odds ratio (OR) for limitations in sports, comparing survivors to siblings, was 5.5 (CI 2.9–10.4; p<0.001). This remained similar after adjusting for family clustering (OR = 5.5; CI 3.0–10.0; p<0.001).

### Limitations on daily activities (physical function (PF) score SF-36)

For every item of the PF score survivors reported more limitations than siblings (**Table 2**), with the biggest discrepancies seen for walking-related activities of daily life and bathing or dressing. Survivors reached a mean PF score of 49.6 (CI 48.9–50.4) compared to a mean of 53.1 (CI 52.5–53.7) in siblings. Adjusting for age and sex, the mean difference was −3.3 (CI −4.5–2.1; p<0.001). Results remained similar (mean difference = −3.3; CI −4.2–2.4; p<0.001) when adjusting for family clustering.

### Survivors diagnosed before and after 1990

We compared results for survivors diagnosed from 1976-89 with those diagnosed 1990–2003 (**Tables S2 and S3**). Prevalence of limitations and differences to siblings remained similar in survivors diagnosed more recently, or tended even to increase. In the first period, 8.8% of survivors and 1.6% of siblings reported a limitation (OR 4.8; CI 2.4–9.6), in the more recent period 10.1% of survivors and 0.6% of siblings (OR 8.3; CI 3.7–18.8; p = 0.025 for effect modification between the two periods).

Mean PF score for daily activities was 50.4 in survivors and 53.5 in siblings of the first period (mean difference −3.1; CI −4.2–1.9), and 48.9 and 52.7, respectively in the second period (mean difference −3.6; CI −5.0–2.1; p = 0.356 for effect modification between the two periods).

When looking only at survivors diagnosed in the last 5 years of our cohort (1998–2003) results were even more pronounced. Among survivors, 12.2% (CI 7.6%–19.0%) reported to suffer from limitations in sporting activities compared to 0.4% of siblings (CI 0.1%–2.4%; OR for age- and sex-adjusted difference between survivors and siblings  = 20.8, CI 6.1–71.2, p<0.001). Survivors diagnosed in the last 5 years had a mean physical function score of 46.4 (CI 43.5–49.4) compared to 52.4 in siblings (CI 51.1–53.7; Coeff. for age- and sex-adjusted mean difference  = −6.0, CI −8.0–4.0, p<0.001; data available from the author).

### Predictors of limitations in sports (survivors only)

**Figure 1:**
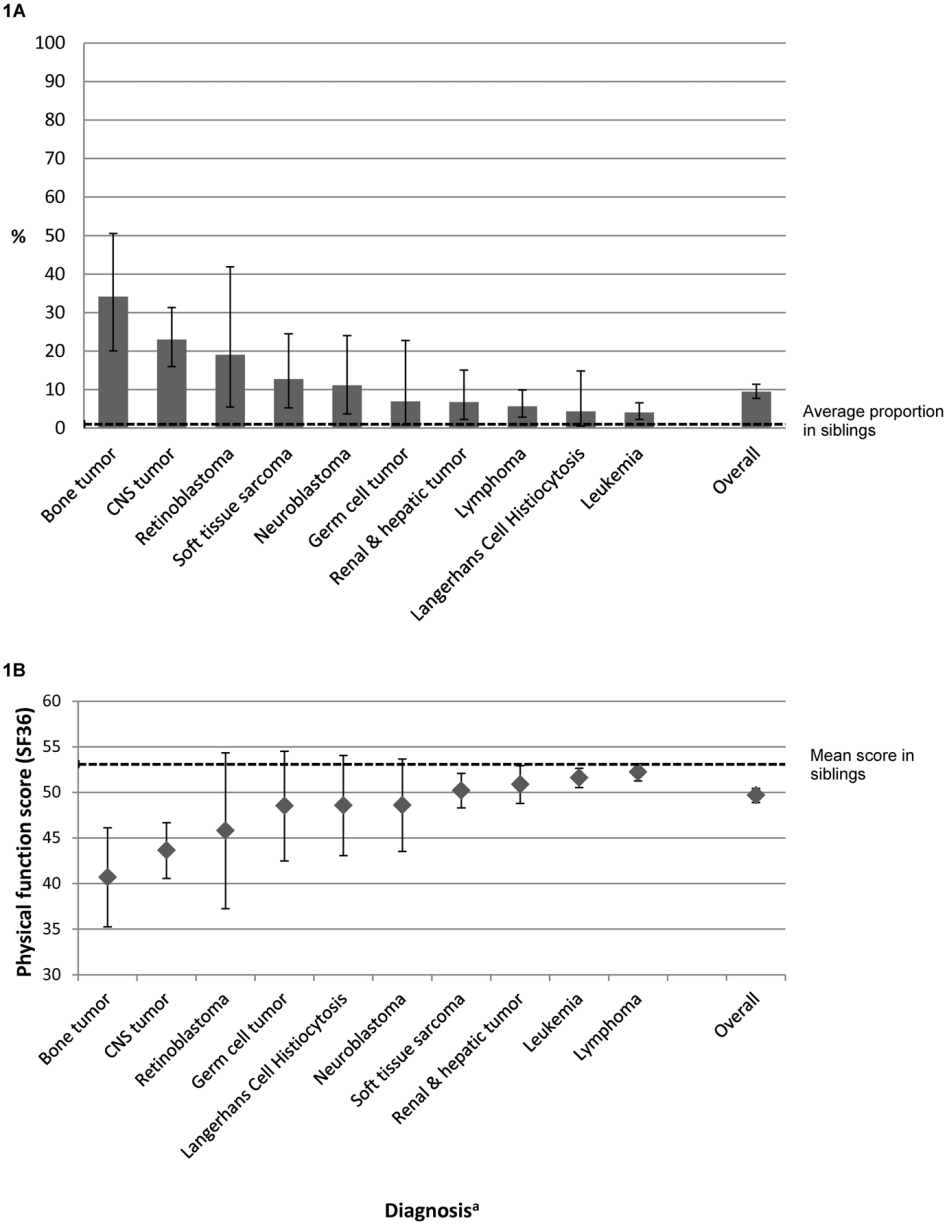
Limitations in sports (proportions) and daily activities (mean SF-36 physical function score) by type of diagnosis. ^a^Diagnosis is classified according to the International Classification of Childhood Cancer – third edition [26]. Abbreviations: CNS, Central Nervous System. *Figure 1A* shows the proportion of survivors reporting a limitation for sporting activities within each diagnostic group. The dotted line indicates the proportion in the sibling controls. *Figure 1B* shows limitations for daily activities (mean scores of the physical function score from the SF-36) stratified by type of diagnosis. Lower scores indicate increased limitations for daily tasks such as walking fast, carry heavy things, move a table, carry grocery bags, climbing stairs, bending down, walking a certain distance, bathing and clothing. The dotted line indicates the mean score of the sibling controls.

**Figure 1A** shows how the proportion of survivors reporting a limitation in sports varied by type of cancer (p<0.001). Survivors of bone tumors were most affected (34% reporting a limitation), followed by survivors of CNS tumors (23%), retinoblastoma (19%), and soft tissue sarcoma (13%).

In the unadjusted regression model (**Table S4**) factors associated with limitations in sports were: having had a CNS tumor (OR 7.1; CI 3.7–13.8), retinoblastoma (OR 5.6; CI 1.7–18.7), bone tumor (OR 12.3; CI 5.4–28.2), or soft tissue sarcoma (OR 3.5; CI 1.4–8.9) and having received radiotherapy (OR 2.1; CI 1.3–3.3). Survivors aged ≥40 years tended to be more limited (p = 0.072). Results from the adjusted regression model (**Table 3**) were comparable, the strongest predictors remaining type of cancer and treatment.

**Table 3:** Predictors of limitations in sports and daily activities (physical function score <45) from two multivariable regression models in survivors.

	Limited in sports						Limited in daily activities				
	%^a^		OR	95% CI		p^c^	%^b^	OR	95% CI		p^c^
*Gender*						0.367					0.158
Male	8.5		1				13.2	1			
Female	10.5		1.23	0.78	1.95		16.7	1.31	0.90	1.90	
*Current age (years)*						0.061					0.496
≤20	10.3		1.13	0.62	2.07		19.0	1.45	0.90	2.32	
20–29.9	9.7		1				13.5	1			
30–39.9	6.5		0.63	0.32	1.22		14.6	1.05	0.64	1.72	
≥40	20.5		2.70	1.02	7.16		15.4	0.97	0.36	2.63	
*Parents education*						0.035					0.008
Primary education	4.6		0.40	0.13	1.18		20.5	1.89	1.03	3.49	
Secondary education	9.8		1				13.7	1			
Tertiary education	8.9		0.98	0.51	1.87		12.6	1.01	0.58	1.76	
Unknown	15.9		2.97	1.19	7.39		26.6	2.80	1.46	5.38	
*Age at diagnosis (years)*						0.964					0.387
<5	8.1		1				14.6	1			
5–9.9	9.7		1.04	0.55	1.97		14.3	0.72	0.43	1.19	
≥10	10.7		1.10	0.56	2.14		15.6	0.92	0.54	1.56	
*Diagnosis (ICCC3 main groups)*						<0.001					<0.001
I Leukemia	4.0		1				9.2	1			
II Lymphoma	5.7		1.16	0.49	2.74		8.2	0.89	0.45	1.75	
III CNS tumor	23.2		9.40	4.26	20.74		30.5	5.76	3.08	10.80	
IV Neuroblastoma	11.1		3.77	1.23	11.55		15.6	2.42	0.95	6.17	
V Retinoblastoma	19.1		8.55	2.26	32.33		19.1	2.88	0.82	10.10	
VI & VII Renal & hepatic tumor^d^	6.8		1.65	0.57	4.80		16.0	2.19	1.04	4.63	
VIII Bone tumor	34.2		13.59	5.55	33.28		45.2	10.87	5.04	23.45	
IX Soft tissue sarcoma	12.7		2.87	1.07	7.70		16.1	1.76	0.77	4.04	
X Germ cell tumor	6.9		2.11	0.44	10.12		10.0	1.15	0.31	4.19	
XI & XII Other tumor^e^	18.2		5.72	1.03	31.76		16.7	1.86	0.34	10.02	
Langerhans Cell Histiocytosis	4.4		1.62	0.35	7.52		15.2	2.91	1.15	7.35	
*Treatment*						<0.001					<0.001
Surgery only	8.2	0.35		0.13	0.90		13.2	0.50	0.23	1.07	
Chemotherapy^f^	7.0	1					10.3	1			
Radiotherapy^g^	13.6	1.61		0.90	2.88		20.6	2.08	1.31	3.32	
Bone marrow transplantation	8.1	0.85		0.22	3.28		25.6	2.98	1.24	7.14	
*Relapse*						0.880					0.326
No	9.3	1					14.2	1			
Yes	11.3	1.06		0.52	2.13		20.6	1.32	0.76	2.29	

Abbreviations: CI, Confidence Interval; CNS, Central Nervous System; ICCC-3, International Classification of Childhood Cancer Third Edition; OR, Odds Ratio.

a Proportion reporting a limitation in sports in each stratum. Column percentages are given.

b Proportion reporting a limitation in daily activities in each stratum. Column percentages are given.

c Global p-values calculated with a likelihood ratio test.

d Hepatic and renal tumors have been merged for this analysis.

e Other malignant epithelial neoplasm, malignant melanoma and other or unspecified malignant neoplasm.

f Chemotherapy may include surgery.

g Radiotherapy may include surgery and/or chemotherapy.

### Predictors of limitations in daily activities (survivors only)

The mean physical function (PF) score also differed by type of cancer (**Figure 1B**), with lowest scores in survivors of bone tumors (mean PF score 40.7), followed by survivors of CNS tumors (43.6) and retinoblastoma (45.8). In the unadjusted model (**Table S4**) factors associated with limitations on daily activities were: low parental education (p = 0.024), having received radiotherapy or bone marrow transplantation (p<0.001) and having suffered from a bone tumor or CNS tumor (p<0.001). Results of the adjusted regression model were similar, showing even stronger associations for type of cancer and treatments (**Table 3**).

## Discussion

This nationwide survey including survivors of all types of childhood cancers diagnosed until 2003 found that survivors were five times more likely than siblings to suffer from limitations in sports and that they had significantly lower physical function scores for activities of daily life. Limitations differed strongly between diagnostic groups, with poorest results for survivors of bone tumors, CNS tumors and retinoblastoma. Importantly, we found no evidence that limitations had decreased in survivors diagnosed recently (1990–2003) and treated according to modern protocols.

### Strengths and limitations

This study has several strengths. It is a representative population-based national cohort study of all Swiss childhood cancer survivors (response rate 78%) as well as their siblings. In contrast to studies from the US, we included all types of childhood cancers, particularly retinoblastoma, a strongly affected group. Our study included survivors from a large age range starting with adolescents, and covered a broad spectrum of activity limitations by assessing both limitations in sports and daily activities. With our open formatted question on the reasons for limitations in sports, we could collect additional information on the underlying causes of limitations in childhood cancer survivors. Finally, a major strength is the wide time period of diagnosis (1976–2003), allowing to evaluate limitations in children diagnosed recently.

The study has also limitations. One is the self-reported assessment of performance limitations, which our study shares with others from the US and UK [17], [18]. We assessed limitations in sporting activities with an open formatted question to identify individual and subjective reasons that keep survivors from being active. Though this might differ from survivors' objective possibilities of physical performance, subjectively experienced limitation is usually more important for practicing sporting activities. Not participating in sporting activities can affect the survivors' health and social contacts [6], [7]. Another limitation is the comparatively low response rate of siblings, making it unclear if the sample is fully representative for the whole sibling population.

### Comparison with other studies

We are not aware of any other studies reporting on limitations in sports of childhood cancer survivors. However, limitations in daily activities have been described by the US childhood cancer survivor study in different contexts [6], [7], [12], [13], [14], [15]. As all results relate to children diagnosed between 1970–1986 they can be compared to the older half of our cohort. One study used the SF-36 and reported mean PF scores of 51.3 in 7147 adult survivors and 55.0 in 388 siblings [15]. This is similar to our findings for children diagnosed before 1990, where mean scores were 50.4 and 53.5 for survivors and siblings, respectively. Comparable to our findings are also differences by type of cancer, with survivors of bone tumors, CNS tumors, Hodgkin's Lymphoma and soft tissue sarcomas scoring lowest. Retinoblastoma patients had not been included in the US study [15].

Another study used the Behavioral Risk Factor Surveillance System Questionnaire (BRFSS) in a sample of 11481 adolescent and adult survivors and 3839 siblings [14], and found more performance limitations in survivors than siblings (OR 1.8, CI 1.7–2.0). Using a different cut-off, Hudson and colleagues found more activity limitations (OR = 2.7) in 9535 adult survivors compared to 2961 siblings [12]. In line with our findings, both studies reported the highest risks in survivors of bone and brain tumors [12], [14].

### Interpretation of the results

Our study confirmed that survivors of childhood cancer have a high risk of physical performance limitations, both in sports and activities of daily life. On physical function score of daily activities survivors scored on average 3.3 points below Siblings. Although this mean difference might not be clinically relevant, it suggests that specific subgroups of survivors may have clinically relevant limitations in daily living. For example survivors of bone tumors on average score 12.4 points below the siblings' mean (**Figure 1B**) and survivors of CNS tumor 9.5 points; both are of clinical relevance.

As a novel finding, we identified underlying disorders, showing that musculoskeletal and neurological problems were most common, followed by pain and fatigue syndromes, weight and endurance problems and cardio-pulmonary symptoms. Limitations were in line with the underlying type of cancer (**Table S1**), such that most survivors of brain tumors reported neurological problems, and survivors of bone tumors reported musculoskeletal problems.

Predictors were similar for limitations in sport and daily activities, underlining the robustness of our results. As expected from previous studies, three diagnostic groups were most strongly affected: survivors of bone tumors, often treated by amputations or other major limb surgery [7], [13], survivors of CNS tumors, with problems of coordination, balance, muscle strength, paralysis, vision or hearing [29], and retinoblastoma patients, suffering from blindness or severe visual impairments [30].

We had hypothesized that improved therapy, such as limb-sparing surgery [31], minimally invasive retinoblastoma therapy [32], and reduction in cranial irradiation might have reduced the risk of physical activity limitations in more recently diagnosed patients. This was not the case in our population. Survivors diagnosed after 1990 had a similar risk for performance limitations as those diagnosed earlier and performed at least as badly compared to age- and sex-adjusted siblings. If anything, they fared worse rather than better. The observed difference between periods of diagnosis may not be clinically relevant. However, our results with their 95% confidence intervals provide strong evidence against an improvement in more recent treatment periods. One possible explanation for a lack of improvement in more recently diagnosed survivors might be that improved therapies result in less severe limitations, but not necessarily less common limitations [30], [33]. Or patients who would not have survived in earlier decades, can nowadays be cured but with a high risk for performance limitations due to an increased burden of therapy. For example the introduction of hematopoietic growth factors or the widespread use of high-dose chemotherapy with autologous stem-cell rescue [34], [35], [36], [37]. Finally, more recently diagnosed survivors might have had less time to cope with their impairments and rate their limitations subjectively different.

### Implication for practice

Survival rates in childhood cancer have improved markedly [2], and it is time to improve quality of survivorship [1]. Quality of life includes independent living and ability to participate in physical activities and social life roles [6], [38]. It is therefore important to preserve functional capacity and reduce the burden of performance limitations in survivors. This can be done by choosing the best therapy, offering physical activity interventions, reducing obesity, developing coping strategies, and enhancing psycho-social well-being [9], [39], [40].

Our results show that main risk factors for performance limitations were type of cancer and subsequent treatment. Thus, at-risk groups are clear, and interventions could start early after or even during therapy. In principle, performance limitations can be avoided or mitigated by primary or secondary prevention strategies.

Primary prevention would be through adaptations of initial therapy. In fact, during the past decades much effort has been put into developing minimally invasive surgical techniques and reducing radiotherapy. Treatment of retinoblastoma with eye-preserving techniques and reduced chemotherapy should lead to better functional outcomes [32], [41]. The same was hoped for limb-sparing surgery for bone tumors. However, first studies did not confirm functionally better results for limb-sparing surgery compared to amputations [33]. In our population, these changes in therapy, which have also been implemented in Switzerland, have not translated to a sizeable reduction in performance limitations. This suggests that there is still room for improvement of minimally invasive therapy techniques and ongoing research is justified and needed.

Secondary prevention is another option for reducing performance limitations and improving quality of survivorship. Many of the common limitations described by survivors in this study, e.g. musculoskeletal and neurological problems, fatigue syndromes, weight, endurance and cardio-pulmonary problems have been shown to respond to interventions during or after treatment [42]. Several studies suggest that physical activity interventions beginning during treatment and continuing during follow-up can reduce performance limitations, improve quality of life, reduce obesity, pain and fatigue and increase independent living status and psychosocial well-being [9], [10], [40], [42], [43], [44], [45], [46]. To encourage lifelong physical activity, exercise programs should be individually tailored, home-based, implemented into daily living, and connected to fun and social contacts [8], [46], [47]. During follow-up consultations physicians but also nursing specialists or physiotherapists could encourage engagement in physical activity and develop personal strategies to include specially needed exercises into daily living. They could for example hand out leaflets with coordinative exercises to be done 10 minutes every day for survivors with neurological problems. Or they could help survivors to find the optimal type of activity and reduce barriers for being active (help for participation in sports club, fitness center, sports class for handicapped, individual sports etc.).

### Conclusion

Despite modern treatments and protocols, survivors of childhood cancer, particularly after bone tumors, brain tumors and retinoblastoma, are at great risk of suffering from performance limitations in sports and activities of daily living. Early, life-long and individually tailored interventions aimed at reducing performance limitations should be implemented in the care of high-risk patients.

## Supporting Information

Figure S1 **Participants and response rate of the Swiss Childhood Cancer Survivor Study.** Figure S1 shows the flow diagram of our study population starting from those eligible to those included in the analysis. ^a^107 survivors without current address include 71 who moved abroad, and 36 who were lost to follow up.
(TIFF)

Table S1 **Description of limitations in sporting activities by type of cancer.**
(DOCX)

Table S2 **Description of limitations in sporting activities and daily activities in survivors diagnosed after 1990 (n = 536) and siblings.** Abbreviations: CI, Confidence Interval; Diff., Difference; N, Number; SF-36, Short Form 36; OR, Odds Ratio. ^a^ Age- and sex-standardized numbers and percentages are given for siblings based on the marginal distribution in survivors. ^b^ OR comparing survivors and siblings in a logistic model adjusting for age and sex. ^c^ P-values calculated from regression models adjusting for age and sex. ^d^ Coefficient comparing mean score in survivors and siblings from linear regression adjusting for age and sex. ^e^ Proportion who indicated to be limited either a lot or a little in single items if the SF-36 physical function score. ^f^ Mean of T-standardized physical function score of the SF-36 (23).
(DOCX)

Table S3 **Description of limitations in sporting activities and daily activities in survivors diagnosed before 1990 (n = 502) and siblings.** Abbreviations: CI, Confidence Interval; Diff., Difference; N, Number; SF-36, Short Form 36; OR, Odds Ratio. ^a^ Age- and sex-standardized numbers and percentages are given for siblings based on the marginal distribution in survivors. ^b^ OR comparing survivors and siblings in a logistic model adjusting for age and sex. ^c^ P-values calculated from regression models adjusting for age and sex. ^d^ Coefficient comparing mean score in survivors and siblings from linear regression adjusting for age and sex. ^e^ Proportion who indicated to be limited either a lot or a little in single items if the SF-36 physical function score. ^f^ Mean of T-standardized physical function score of the SF-36 (23).
(DOCX)

Table S4 **Predictors of limitations in sports and daily activities (physical function score <45) from univariable regression models in survivors.** Abbreviations: CI, Confidence Interval; CNS, Central Nervous System; ICCC-3, International Classification of Childhood Cancer Third Edition; OR, Odds Ratio. ^a^ Proportion reporting a limitation in sports in each stratum. Column percentages are given. ^b^ Proportion reporting a limitation in daily activities in each stratum. Column percentages are given. ^c^ Global p-values calculated with a likelihood ration test. ^d^ Hepatic and renal tumors have been merged for this analysis. ^e^ Other malignant epithelial neoplasm, malignant melanoma and other or unspecified malignant neoplasm. ^f^ Chemotherapy may include surgery. ^g^ Radiotherapy may include surgery and/or chemotherapy.
(DOCX)
