# Supplementary material for: Physical Performance Limitations in Adolescent and Adult Survivors of Childhood Cancer and Their Siblings
Source: PLoS One. 2012 Oct 17;7(10):e47944. doi: 10.1371/journal.pone.0047944 (PMC3474773; doi:10.1371/journal.pone.0047944)
Supplement: Table S1 — Description of limitations in sporting activities by type of cancer. (DOCX) [file pone.0047944.s002.docx]

**Table S1: Description of limitations in sporting activities by type of cancer**

|  | **Type of reported limitation** | **N** | **%** |
| --- | --- | --- | --- |
| *Leukemia* | | | |
|  | No limitation | 357 | 96.0 |
|  | Musculoskeletal problems | 7 | 1.9 |
|  | Neurological problems | 4 | 1.1 |
|  | Pain- and fatigue syndromes | 2 | 0.5 |
|  | Weight and endurance problems | 1 | 0.3 |
|  | Psychological problems | 1 | 0.3 |
|  | Total | 372 | 100 |
| *Lymphoma* | | | |
|  | No limitation | 183 | 94.3 |
|  | Musculoskeletal problems | 9 | 4.6 |
|  | Cardio-pulmonary problems | 2 | 1.0 |
|  | Total | 194 | 100 |
| *CNS tumor* | | | |
|  | No limitation | 97 | 77.0 |
|  | Musculoskeletal problems | 5 | 4.0 |
|  | Neurological problems | 18 | 14.3 |
|  | Pain- and fatigue syndromes | 3 | 2.4 |
|  | Weight and endurance problems | 1 | 0.8 |
|  | Visual impairment | 1 | 0.8 |
|  | Psychological problems | 1 | 0.8 |
|  | Total | 126 | 100 |
| *Neuroblastoma* | | | |
|  | No limitation | 40 | 88.9 |
|  | Musculoskeletal problems | 1 | 2.2 |
|  | Neurological problems | 1 | 2.2 |
|  | Weight and endurance problems | 1 | 2.2 |
|  | Problem unknown | 2 | 4.4 |
|  | Total | 45 | 100 |
| *Retinoblastoma* | | | |
|  | No limitation | 17 | 81.0 |
|  | Neurological problems | 1 | 4.8 |
|  | Visual impairment | 1 | 4.8 |
|  | Problem unknown | 2 | 9.5 |
|  | Total | 21 | 100 |
| *Renal & hepatic tumor* | | | |
|  | No limitation | 69 | 93.2 |
|  | Musculoskeletal problems | 3 | 4.1 |
|  | Weight and endurance problems | 1 | 1.4 |
|  | Visual impairment | 1 | 1.4 |
|  | Total | 74 | 100 |
| *Bone tumors* | | | |
|  | No limitation | 27 | 65.9 |
|  | Musculoskeletal problems | 13 | 31.7 |
|  | Problem unknown | 1 | 2.4 |
|  | Total | 41 | 100 |
| *Soft tissue sarcoma* | | | |
|  | No limitation | 48 | 87.3 |
|  | Musculoskeletal problems | 3 | 5.4 |
|  | Neurological problems | 2 | 3.6 |
|  | Cardio-pulmonary problems | 1 | 1.8 |
|  | Pain- and fatigue syndromes | 1 | 1.8 |
|  | Total | 55 | 100 |
| *Germ cell tumor* | | | |
|  | No limitation | 27 | 93.1 |
|  | Pain- and fatigue syndromes | 1 | 3.5 |
|  | Problem unknown | 1 | 3.5 |
|  | Total | 29 | 100 |
| *Other tumor* | | | |
|  | No limitation | 9 | 81.8 |
|  | Musculoskeletal problems | 1 | 9.1 |
|  | Weight and endurance problems | 1 | 9.1 |
|  | Total | 11 | 100 |
| *Langerhans cell histiocytosis* | | | |
|  | No limitation | 44 | 95.7 |
|  | Musculoskeletal problems | 1 | 2.2 |
|  | Neurological problems | 1 | 2.2 |
|  | Total | 46 | 100 |
