# Supplementary material for: Physical Performance Limitations in Adolescent and Adult Survivors of Childhood Cancer and Their Siblings
Source: PLoS One. 2012 Oct 17;7(10):e47944. doi: 10.1371/journal.pone.0047944 (PMC3474773; doi:10.1371/journal.pone.0047944)
Supplement: Table S2 — Description of limitations in sporting activities and daily activities in survivors diagnosed after 1990 (n = 536) and siblings. Abbreviations: CI, Confidence Interval; Diff., Difference; N, Number; SF-36, Short Form 36; OR, Odds Ratio. a Age- and sex-standardized numbers and percentages are given for siblings based on the marginal distribution in survivors. b OR comparing survivors and siblings in a logistic model adjusting for age and sex. c P-values calculated from regression models adjusting for age and sex. d Coefficient comparing mean score in survivors and siblings from linear regression adjusting for age and sex. e Proportion who indicated to be limited either a lot or a little in single items if the SF-36 physical function score. f Mean of T-standardized physical function score of the SF-36 (23). (DOCX) [file pone.0047944.s003.docx]

**Table S2: Description of limitations in sporting activities and daily activities in survivors diagnosed after 1990 (n=536) and siblings**

|  | **Survivors diagnosed ≥1990** | | |  | **Siblings^a^** | | |  | | | |
| --- | --- | --- | --- | --- | --- | --- | --- | --- | --- | --- | --- |
| **Limitation in sporting activities** |  |  |  |  |  |  |  |  |  |  |  |
| *Medical conditions* | **N** | **%** | **95% CI** |  | **N** | **%** | **95% CI** |  | **OR^b^** | **95% CI** | **p-value^c^** |
| Musculoskeletal problems | 23 | 4.5 | 3.0-6.6 |  | 0 | 0.02 | 0.002-0.1 |  |  |  |  |
| Neurological problems | 13 | 2.5 | 1.5-4.3 |  | 1 | 0.2 | 0.02-1.0 |  |  |  |  |
| Pain and fatigue syndromes | 6 | 1.2 | 0.5-2.6 |  | 0 | 0 | - |  |  |  |  |
| Weight and endurance problems | 2 | 0.4 | 0.1-1.5 |  | 0 | 0 | - |  |  |  |  |
| Cardio-pulmonary problems | 2 | 0.4 | 0.1-1.5 |  | 0.5 | 0.1 | 0.02-1.0 |  |  |  |  |
| Visual impairment | 2 | 0.4 | 0.1-1.5 |  | 0 | 0 | - |  |  |  |  |
| Psychological problems | 2 | 0.4 | 0.1-1.5 |  | 0 | 0 | - |  |  |  |  |
| Problem unknown | 2 | 0.4 | 0.1-1.5 |  | 1.5 | 0.3 | 0.04-2.3 |  |  |  |  |
| **Total proportion** | **52** | **10.1** | **7.8-13.0** |  | **3** | **0.6** | **0.2-2.1** |  | **8.3^b^** | **3.7-18.8** | **<0.001** |
|  |  |  |  |  |  |  |  |  |  |  |  |
| **Limitations in daily activities** |  |  |  |  |  |  |  |  |  |  |  |
| *Items of physical function score* | **N** | **%** | **95% CI** |  | **N** | **%** | **95% CI** |  | **Diff.^d^** | **95% CI** | **p-value^c^** |
| Vigorous activities | 173 | 32.5^e^ | 28.6-36.6 |  | 81 | 15.2^e^ | 11.6-19.7 |  |  |  |  |
| Moderate activities | 52 | 9.7^e^ | 7.5-12.6 |  | 14 | 2.7^e^ | 1.3-5.2 |  |  |  |  |
| Carrying groceries | 41 | 7.7^e^ | 5.7-10.3 |  | 19 | 3.6^e^ | 2.0-6.4 |  |  |  |  |
| Climbing several flights of stairs | 55 | 10.3^e^ | 8.0-13.8 |  | 23 | 4.3^e^ | 2.5-7.4 |  |  |  |  |
| Climbing one flight of stairs | 19 | 3.6^e^ | 2.3-5.5 |  | 5 | 1.0^e^ | 0.3-3.4 |  |  |  |  |
| Bending down | 64 | 12.0^e^ | 9.5-15.0 |  | 26 | 4.9^e^ | 2.7-8.6 |  |  |  |  |
| Walking more than 1 kilometer | 55 | 10.3^e^ | 8.0-13.2 |  | 14 | 2.7^e^ | 1.3-5.6 |  |  |  |  |
| Walking several 100 meters | 31 | 5.8^e^ | 4.1-8.1 |  | 6 | 1.2^e^ | 0.4-3.5 |  |  |  |  |
| Walking 100 meters | 18 | 3.4^e^ | 2.1-5.3 |  | 4 | 0.7^e^ | 0.2-2.7 |  |  |  |  |
| Bathing or dressing | 16 | 3.0^e^ | 1.8-4.8 |  | 4 | 0.7^e^ | 0.2-2.6 |  |  |  |  |
| **Physical function score (mean)** | | **48.9^f^** | **47.8-50.1** |  |  | **52.7^f^** | **51.7-53.6** |  | **-3.6^d^** | **-5.0--2.1** | **<0.001** |

^a^ Age- and sex-standardized numbers and percentages are given for siblings based on the marginal distribution in survivors.

^b^ OR comparing survivors and siblings in a logistic model adjusting for age and sex.

^c^ P-values calculated from regression models adjusting for age and sex.

^d^ Coefficient comparing mean score in survivors and siblings from linear regression adjusting for age and sex.

^e^ Proportion who indicated to be limited either a lot or a little in single items if the SF-36 physical function score.

^f^ Mean of T-standardized physical function score of the SF-36 (23).

Abbreviations: CI, Confidence Interval; Diff., Difference; N, Number; SF-36, Short Form 36; OR, Odds Ratio.
