# Supplementary material for: Physical Performance Limitations in Adolescent and Adult Survivors of Childhood Cancer and Their Siblings
Source: PLoS One. 2012 Oct 17;7(10):e47944. doi: 10.1371/journal.pone.0047944 (PMC3474773; doi:10.1371/journal.pone.0047944)
Supplement: Table S3 — Description of limitations in sporting activities and daily activities in survivors diagnosed before 1990 (n = 502) and siblings. Abbreviations: CI, Confidence Interval; Diff., Difference; N, Number; SF-36, Short Form 36; OR, Odds Ratio. a Age- and sex-standardized numbers and percentages are given for siblings based on the marginal distribution in survivors. b OR comparing survivors and siblings in a logistic model adjusting for age and sex. c P-values calculated from regression models adjusting for age and sex. d Coefficient comparing mean score in survivors and siblings from linear regression adjusting for age and sex. e Proportion who indicated to be limited either a lot or a little in single items if the SF-36 physical function score. f Mean of T-standardized physical function score of the SF-36 (23). (DOCX) [file pone.0047944.s004.docx]

**Table S3: Description of limitations in sporting activities and daily activities in survivors diagnosed before 1990 (n=502) and siblings**

|  | **Survivors diagnosed <1990** | | |  | **Siblings^a^** | | |  | | | |
| --- | --- | --- | --- | --- | --- | --- | --- | --- | --- | --- | --- |
| **Limitation in sporting activities** |  |  |  |  |  |  |  |  |  |  |  |
| *Medical conditions* | **N** | **%** | **95% CI** |  | **N** | **%** | **95% CI** |  | **OR^b^** | **95% CI** | **p-value^c^** |
| Musculoskeletal problems | 20 | 4.0 | 2.6-6.2 |  | 5 | 0.9 | 0.002-0.1 |  |  |  |  |
| Neurological problems | 14 | 2.8 | 1.7-4.7 |  | 3 | 0.5 | 0.2-1.6 |  |  |  |  |
| Pain and fatigue syndromes | 1 | 0.2 | 0.03-1.4 |  | 0 | 0 | - |  |  |  |  |
| Weight and endurance problems | 3 | 0.6 | 0.2-1.9 |  | 0 | 0 | - |  |  |  |  |
| Cardio-pulmonary problems | 1 | 0.2 | 0.03-1.4 |  | 1 | 0.2 | 0.02-1.2 |  |  |  |  |
| Visual impairment | 1 | 0.2 | 0.03-1.4 |  | 0 | 0 | - |  |  |  |  |
| Psychological problems | 0 | 0 | - |  | 0 | 0 | - |  |  |  |  |
| Problem unknown | 4 | 0.8 | 0.3-2.1 |  | 0 | 0 | 0.04-2.3 |  |  |  |  |
| **Total proportion** | **44** | **8.8** | **6.6-11.7** |  | **9** | **1.6** | **0.8-3.1** |  | **4.8^b^** | **2.4-9.6** | **<0.001** |
|  |  |  |  |  |  |  |  |  |  |  |  |
| **Limitations in daily activities** |  |  |  |  |  |  |  |  |  |  |  |
| *Items of physical function score* | **N** | **%** | **95% CI** |  | **N** | **%** | **95% CI** |  | **Diff.^d^** | **95% CI** | **p-value^c^** |
| Vigorous activities | 163 | 32.8^e^ | 28.8-37.1 |  | 103 | 19.3^e^ | 15.8-23.5 |  |  |  |  |
| Moderate activities | 39 | 7.9^e^ | 5.8-10.6 |  | 15 | 2.8^e^ | 1.6-4.7 |  |  |  |  |
| Carrying groceries | 41 | 8.3^e^ | 5.2-11.1 |  | 19 | 3.6^e^ | 2.3-5.7 |  |  |  |  |
| Climbing several flights of stairs | 53 | 10.8^e^ | 8.3-13.8 |  | 19 | 3.6^e^ | 2.2-5.8 |  |  |  |  |
| Climbing one flight of stairs | 15 | 3.0^e^ | 1.8-5.0 |  | 23 | 4.4^e^ | 1.1-1.8 |  |  |  |  |
| Bending down | 60 | 12.2^e^ | 9.5-15.3 |  | 34 | 6.3^e^ | 4.2-9.2 |  |  |  |  |
| Walking more than 1 kilometer | 43 | 8.7^e^ | 6.5-11.5 |  | 10 | 1.9^e^ | 0.9-3.7 |  |  |  |  |
| Walking several 100 meters | 26 | 5.3^e^ | 3.6-7.6 |  | 4 | 0.7^e^ | 0.3-2.0 |  |  |  |  |
| Walking 100 meters | 20 | 4.1^e^ | 2.6-6.2 |  | 5 | 0.9^e^ | 0.4-2.3 |  |  |  |  |
| Bathing or dressing | 13 | 2.6^e^ | 1.5-4.5 |  | 4 | 0.8^e^ | 0.3-2.2 |  |  |  |  |
| **Physical function score (mean)** | | **50.4^f^** | **49.4-51.5** |  |  | **53.5^f^** | **53.0-54.0** |  | **-3.1^d^** | **-4.2--1.9** | **<0.001** |

^a^ Age- and sex-standardized numbers and percentages are given for siblings based on the marginal distribution in survivors.

^b^ OR comparing survivors and siblings in a logistic model adjusting for age and sex.

^c^ P-values calculated from regression models adjusting for age and sex.

^d^ Coefficient comparing mean score in survivors and siblings from linear regression adjusting for age and sex.

^e^ Proportion who indicated to be limited either a lot or a little in single items if the SF-36 physical function score.

^f^ Mean of T-standardized physical function score of the SF-36 (23).

Abbreviations: CI, Confidence Interval; Diff., Difference; N, Number; SF-36, Short Form 36; OR, Odds Ratio.
