# Supplementary material for: Physical Performance Limitations in Adolescent and Adult Survivors of Childhood Cancer and Their Siblings
Source: PLoS One. 2012 Oct 17;7(10):e47944. doi: 10.1371/journal.pone.0047944 (PMC3474773; doi:10.1371/journal.pone.0047944)
Supplement: Table S4 — Predictors of limitations in sports and daily activities (physical function score <45) from univariable regression models in survivors. Abbreviations: CI, Confidence Interval; CNS, Central Nervous System; ICCC-3, International Classification of Childhood Cancer Third Edition; OR, Odds Ratio. a Proportion reporting a limitation in sports in each stratum. Column percentages are given. b Proportion reporting a limitation in daily activities in each stratum. Column percentages are given. c Global p-values calculated with a likelihood ration test. d Hepatic and renal tumors have been merged for this analysis. e Other malignant epithelial neoplasm, malignant melanoma and other or unspecified malignant neoplasm. f Chemotherapy may include surgery. g Radiotherapy may include surgery and/or chemotherapy. (DOCX) [file pone.0047944.s005.docx]

Table S4: Predictors of limitations in sports and daily activities (physical function score <45) from univariable regression models in survivors

|  | |  | **Limited in sports** | | | | | | |  | **Limited in daily activities** | | | | | | | | |
| --- | --- | --- | --- | --- | --- | --- | --- | --- | --- | --- | --- | --- | --- | --- | --- | --- | --- | --- | --- |
|  |  |  | **%**^a^ |  | **OR** | **95% CI** | | | **p**^c^ |  | **%**^b^ |  | **OR** | **95% CI** | | | **p**^c^ | |  |
| *Gender* | |  |  |  |  |  |  |  | 0.275 |  |  |  |  |  |  |  | | 0.111 | |
|  | Male |  | 8.5 |  | 1 |  |  |  |  |  | 13.2 |  | 1 |  |  |  | |  | |
|  | Female |  | 10.5 |  | 1.26 | 0.83 | - | 1.93 |  |  | 16.7 |  | 1.32 | 0.94 | - | 1.86 | |  | |
| *Current age (years)* | |  |  |  |  |  |  |  | 0.072 |  |  |  |  |  |  |  | | 0.319 | |
|  | ≤ 20 |  | 10.2 |  | 1.07 | 0.62 | - | 1.85 |  |  | 18.9 |  | 1.51 | 0.99 | - | 2.30 | |  | |
|  | 20-29.9 |  | 9.6 |  | 1 |  |  |  |  |  | 13.4 |  | 1 |  |  |  | |  | |
|  | 30-39.9 |  | 6.5 |  | 0.65 | 0.35 | - | 1.19 |  |  | 14.6 |  | 1.10 | 0.71 | - | 1.72 | |  | |
|  | ≥ 40 |  | 20.5 |  | 2.43 | 1.06 | - | 5.54 |  |  | 15.4 |  | 1.17 | 0.48 | - | 2.89 | |  | |
| *Education of parents* | |  |  |  |  |  |  |  | 0.179 |  |  |  |  |  |  |  | | 0.024 | |
|  | Primary education |  | 4.6 |  | 0.44 | 0.16 | - | 1.24 |  |  | 20.5 |  | 1.63 | 0.93 | - | 2.85 | |  | |
|  | Secondary education |  | 9.8 |  | 1 |  |  |  |  |  | 13.6 |  | 1 |  |  |  | |  | |
|  | Tertiary education |  | 8.8 |  | 0.89 | 0.49 | - | 1.62 |  |  | 12.5 |  | 0.90 | 0.54 | - | 1.51 | |  | |
|  | Unknown |  | 15.9 |  | 1.74 | 0.75 | - | 4.05 |  |  | 26.6 |  | 2.29 | 1.26 | - | 4.15 | |  | |
| *Age at diagnosis (years)* | |  |  |  |  |  |  |  | 0.478 |  |  |  |  |  |  |  | | 0.892 | |
|  | < 5 |  | 8.1 |  | 1 |  |  |  |  |  | 14.5 |  | 1 |  |  |  | |  | |
|  | 5-9.9 |  | 9.7 |  | 1.23 | 0.71 | - | 2.13 |  |  | 14.3 |  | 0.99 | 0.64 | - | 1.53 | |  | |
|  | ≥ 10 |  | 10.6 |  | 1.36 | 0.82 | - | 2.24 |  |  | 15.5 |  | 1.08 | 0.73 | - | 1.62 | |  | |
| *Diagnosis (ICCC-3 main groups)* | |  |  |  |  |  |  |  | <0.001 |  |  |  |  |  |  |  | | <0.001 | |
|  | I Leukemia |  | 4.0 |  | 1 |  |  |  |  |  | 9.1 |  | 1 |  |  |  | |  | |
|  | II Lymphoma |  | 5.7 |  | 1.43 | 0.64 | - | 3.18 |  |  | 8.2 |  | 0.89 | 0.48 | - | 1.65 | |  | |
|  | III CNS tumor |  | 23.0 |  | 7.12 | 3.67 | - | 13.80 |  |  | 30.3 |  | 4.32 | 2.60 | - | 7.19 | |  | |
|  | IV Neuroblastoma |  | 11.1 |  | 2.98 | 1.03 | - | 8.62 |  |  | 15.6 |  | 1.83 | 0.76 | - | 4.41 | |  | |
|  | V Retinoblastoma |  | 19.1 |  | 5.60 | 1.68 | - | 18.69 |  |  | 19.1 |  | 2.34 | 0.75 | - | 7.34 | |  | |
|  | VI & VII Renal & hepatic tumor^d^ |  | 6.8 |  | 1.72 | 0.61 | - | 4.90 |  |  | 16.0 |  | 1.89 | 0.93 | - | 3.85 | |  | |
|  | VIII Bone tumor |  | 34.2 |  | 12.34 | 5.40 | - | 28.21 |  |  | 45.2 |  | 8.21 | 4.08 | - | 16.54 | |  | |
|  | IX Soft tissue sarcoma |  | 12.7 |  | 3.47 | 1.35 | - | 8.94 |  |  | 16.1 |  | 1.90 | 0.86 | - | 4.21 | |  | |
|  | X Germ cell tumor |  | 6.9 |  | 1.76 | 0.38 | - | 8.11 |  |  | 10.0 |  | 1.10 | 0.32 | - | 3.83 | |  | |
|  | XI & XII Other tumor^e^ |  | 18.2 |  | 5.29 | 1.05 | - | 26.64 |  |  | 16.7 |  | 1.99 | 0.42 | - | 9.44 | |  | |
|  | Langerhans Cell Histiocytosis |  | 4.4 |  | 1.08 | 0.24 | - | 4.89 |  |  | 14.9 |  | 1.74 | 0.73 | - | 4.17 | |  | |
| *Treatment* | |  |  |  |  |  |  |  | 0.014 |  |  |  |  |  |  |  | | <0.001 | |
|  | Surgery only |  | 8.2 |  | 1.19 | 0.56 | - | 2.56 |  |  | 13.2 |  | 1.32 | 0.71 | - | 2.43 | |  | |
|  | Chemotherapy^f^ |  | 7.0 |  | 1 |  |  |  |  |  | 10.3 |  | 1 |  |  |  | |  | |
|  | Radiotherapy^g^ |  | 13.6 |  | 2.10 | 1.33 | - | 3.32 |  |  | 20.6 |  | 2.25 | 1.54 | - | 3.29 | |  | |
|  | Bone marrow transplantation |  | 8.1 |  | 1.18 | 0.35 | - | 4.03 |  |  | 25.6 |  | 2.99 | 1.38 | - | 6.48 | |  | |
| *Relapse* | |  |  |  |  |  |  |  | 0.492 |  |  |  |  |  |  |  | | 0.091 | |
|  | No |  | 9.3 |  | 1 |  |  |  |  |  | 14.2 |  | 1 |  |  |  | |  | |
|  | Yes |  | 11.3 |  | 1.25 | 0.66 | - | 2.38 |  |  | 20.6 |  | 1.57 | 0.95 | - | 2.59 | |  | |

^a^ Proportion reporting a limitation in sports in each stratum. Column percentages are given.

^b^ Proportion reporting a limitation in daily activities in each stratum. Column percentages are given.

^c^ Global p-values calculated with a likelihood ration test.

^d^ Hepatic and renal tumors have been merged for this analysis.

^e^ Other malignant epithelial neoplasm, malignant melanoma and other or unspecified malignant neoplasm.

^f^ Chemotherapy may include surgery.

^g^ Radiotherapy may include surgery and/or chemotherapy.

Abbreviations: CI, Confidence Interval; CNS, Central Nervous System; ICCC-3, International Classification of Childhood Cancer Third Edition; OR, Odds Ratio.
